# Call to Action: Creating Resources for Radiology Technologists to Capture Higher Quality Portable Chest X-rays

**DOI:** 10.7759/cureus.29197

**Published:** 2022-09-15

**Authors:** Michael X Jin, Kevin Gilotra, Austin Young, Elaine Gould

**Affiliations:** 1 Radiology, Stony Brook University Hospital, Stony Brook, USA

**Keywords:** medical education, imaging quality, medical errors, imaging, medical quality, radiology technologists, covid-19, pcxr, portable chest x-rays, radiology

## Abstract

Background

Patient rotation, foreign body overlying anatomy, and anatomy out of field of view can have detrimental impacts on the diagnostic quality of portable chest x-rays (PCXRs), especially as the number of PCXR imaging increases due to the coronavirus disease 2019 (COVID-19) pandemic. Although preventable, these “quality failures” are common and may lead to interpretative and diagnostic errors for the radiologist.

Aims

In this study, we present a baseline quality failure rate of PCXR imaging as observed at our institution. We also conduct a focus group highlighting the key issues that lead to the problematic images and discuss potential interventions targeting technologists that can be implemented to address imaging quality failure rate.

Materials and methods

A total of 500 PCXRs for adult patients admitted to a large university hospital between July 12, 2021, and July 25, 2021, were obtained for evaluation of quality. The PCXRs were evaluated by radiology residents for failures in technical image quality. The images were categorized into various metrics including the degree of rotation and obstruction of anatomical structures. After collecting the data, a focus group involving six managers of the technologist department at our university hospital was conducted to further illuminate the key barriers to quality PCXRs faced at our institution..

Results

Out of the 500 PCXRs evaluated, 231 were problematic (46.2%). 43.5% of the problematic films with a repeat PCXR within one week showed that there was a technical problem impacting the ability to detect pathology. Most problematic films also occurred during the night shift (48%). Key issues that lead to poor image quality included improper patient positioning, foreign objects covering anatomy, and variances in technologists' training. Three interventions were proposed to optimize technologist performance that can lower quality failure rates of PCXRs. These include a longitudinal educational curriculum involving didactic sessions, adding nursing support to assist technologists, and adding an extra layer of verification by internal medicine residents before sending the films to the radiologist. The rationale for these interventions is discussed in detail so that a modified version can be implemented in other hospital systems.

Conclusion

This study illustrates the high baseline error rate in image quality of PCXRs at our institution and demonstrates the need to improve on image quality. Poor image quality negatively impacts the interpretive accuracy of radiologists and therefore leads to wrong diagnoses. Increasing educational resources and support for technologists can lead to higher image quality and radiologist accuracy.

## Introduction

Portable chest x-rays (PCXRs) have become widely used in hospitals across the United States, not only for patients who are too sick to be transferred to the radiology department but also for routine floor patients [1]. The importance of PCXRs has only increased during the coronavirus disease 2019 (COVID-19) pandemic. Compared to imaging requests prior to the COVID-19 pandemic, studies have shown a near 70% increased demand for PCXRs [2]. The greater volume of imaging requests has made it increasingly difficult for technologists to consistently capture high-quality films for radiologists to interpret [3]. This can be a result of increased fatigue, decreased job satisfaction, and less time for technologists to communicate with radiologists. Combined, these factors may lead to an increase in diagnostic errors [4]. This is because a radiologist’s interpretive accuracy is mediated by the technical quality of the film, including the precision of patient positioning, the quality of relationships they have with their technologists, and the effectiveness of communication between the radiologist and technologist [5].

While the results of accurate patient positioning are easy to understand, the benefits of strong relationships and effective communication strategies may be less obvious. For example, when radiologists receive a problematic film, they must consult technologists to determine why the PCXR produced this result in order to reach a proper diagnosis [6]. Problematic films can be caused by obstructed anatomy, severe patient rotation, or foreign objects that cover underlying pathology [7]. In any of these situations, the radiologist and technologist must troubleshoot together to determine the problem; however, there may not be enough time for adequate communication between the technologist and radiologist as the number of imaging requests continue to climb [8]. Furthermore, in larger hospital imaging departments, technologists and radiologists may not even have an effective means of instant communication with each other to troubleshoot problematic imaging [9]. To further complicate the issue, each technologist has varying abilities in capturing PCXR films depending on years of practice and the imaging device they operate [10]. Radiologists interpreting films from multiple technologists with varying abilities may see significant variations in patient positioning and maladaptive rotation between individual PCXRs, which may compromise the accuracy of the PCXR interpretation [11]. Providing technologists with more hospital resources and education could prove beneficial to help improve image quality and therefore diagnostic accuracy.

To improve image quality, two potential solutions have been proposed in the current literature that involves increasing the number of available resources for technologists. The first solution is to simply hire a greater number of technologists to lower the workload per technologist [12,13]. The theory behind this is two-fold. More staff can help improve communication with radiologists while facilitating the workload of technologists as they handle increased imaging requests [6,14]. However, hiring more personnel can also drain the finances of the radiology department and become unsustainable quickly. It could also make the system significantly more complex because there would be more individual operators being delegated one task [9]. Another potential solution is implementing a more conservative approach in which PCXRs are only ordered in situations where they are absolutely needed. Although this would decrease the workload for technologists, this can result in many missed diagnoses [15,16]. Additionally, radiologists would have fewer total films for comparison with each individual patient, resulting in even more missed diagnoses [15,16]. Although more floor staff and fewer imaging requests are solutions that may work well in theory, there are many drawbacks that make implementing them in a hospital setting difficult.

Current studies analyzing mammography and ultrasound have shown that technologist performance directly impacts the radiologist's interpretative performance [5]. Therefore, a different approach directly involving optimizing technologist performance becomes increasingly attractive. By optimizing technologist performance, hospital systems can ensure that each patient receives high-quality imaging and an accurate image interpretation from the radiologist without the drawbacks such as cost and missed diagnoses. Optimizing technologist performance is a multi-faceted effort but can involve increasing the number of available resources, educational opportunities, and support for the technologists. Additionally, having technologists perform only one type of imaging has been shown to be linked with better clinical performance [5].

This paper demonstrates the importance of creating resources and systems for technologists to capture high-quality PCXRs and proposes interventions that can optimize technologist performance. We evaluate the current state of PCXR quality at our hospital system and present a baseline report on the rate our hospital system produces problematic films. We evaluate the key issues that lead to the problematic images specific to our institution. Finally, we discuss potential interventions that can be implemented to address the high baseline quality failure rate observed in our recently captured PCXRs. Most importantly, this paper rationalizes the presented interventions so that a modified version can be implemented in other hospital systems.

## Materials and methods

Part 1

A total of 500 PCXRs for adult patients admitted to Stony Brook University Hospital, Stony Brook, New York, between July 12, 2021, and July 25, 2021, were obtained for the evaluation of quality. To determine baseline error rates made by radiology technologists, PCXRs were evaluated by radiology residents for failures in technical image quality that degrades the interpretive quality of films. These factors were categorized into: (i) significant patient horizontal rotation, (ii) significant patient vertical rotation, (iii) partial or complete obstruction of anatomical structures by overlying body parts or foreign bodies, and/or (iv) absence of anatomical structures (Table 1). To better understand potential causes of technical errors made by hospital floor staff, the number of quality failures during various shift timings was evaluated. The subcategories were “Overnight” shift, “Morning” shift, and “Evening” shift, which corresponded with shift timings of 12:00 AM - 7:59 AM, 8:00 AM - 3:59 PM, and 4:00 PM - 11:59 PM, respectively, noted in Table 1. 

**Table 1:** Questionnaire completed by the radiology residents responsible for each of the 500 PCXRs that were interpreted before and after the intervention. PCXRs: portable chest x-rays

Question	Range of Responses
1. Does the anatomy of the image suggest there is patient rotation?	0: Almost none
	1: Mild rotation (clavicles appear asymmetric or one clavicle appears higher than the other)
	2: Subjective rotation
	3: Severe rotation (the sternum and spinous process no longer overlap)
2. Does the anatomy of the image suggest there is vertical or up/down rotation?	0: No
	1: Yes (clavicles and second posterior thoracic rib no longer overlap, or clavicles are positioned below the third posterior rib border)
3. Are there any foreign or external objects overlying the chest that either obscure significant amounts of anatomy or obscure critical areas (e.g. lung apices for pneumothorax and costophrenic angles for pleural effusions)?	0: No
	1: Yes
4. Any portion of chest anatomy cut-off or obscured? Please specify.	0: No
	1: Yes (specifications: costophrenic angle, first ribs, lateral ribs, lateral lung, lung apices, lung bases)
5. Did the technical problem impact ability to detect finding when compared with a prior study or future study?	0: No
	1: Yes
6. During what shift was this image acquired?	1: Morning Shift (8:00 AM to 3:59 PM)
	2: Evening Shift (4:00 PM to 11:59 PM)
	3: Night Shift (12:00 AM to 7:59 AM)
7. Which service was this study taken from?	1: Medicine
	2: Surgery
	3: Intensive Cardiac Rehabilitation (ICR) or Cardiac Acute Care Unit (CACU)
	4: Intensive Care Unit (ICU)
	5: Emergency Department

Part 2

After collecting the data, a focus group involving six managers of the technologist department at our university hospital was created. This focus group involved a discussion between the technologist managers and resident/attending radiologists illuminating the key barriers to quality PCXRs specifically faced at our university. Additionally, to subsequently address the high level of baseline error rates made by technologists, possible interventions targeting technologist performance were discussed. Finally, the rationale for these interventions was discussed and presented so that a modified version can be implemented in other hospital systems.

## Results

The results of the baseline error rates at our institution are presented in Table 2. Out of the 500 PCXRs examined, 231 were problematic with a mean patient rotation score of 1.3. The patient rotation score was generated by assessing the degree of rotation, in which mild rotation received a score of 1, subjective rotation received a score of 2, and severe rotation received a score of 3. This data was further stratified to reveal that 29.9% of those problematic films were mildly rotated, 30.7% were subjectively rotated, and 13.4% were severely rotated. The number of PCXRs with up/down rotation was 43.2%, the number of PCXRs with external or foreign objects obscuring anatomy was 50.2%, and the number of PCXRs with partial or complete anatomy absent was 49.8%. Out of the 231 problematic films, 168 had a repeat study within one week to compare. Out of those 168, 43.5% resulted in a technical problem impacting the ability to detect pathology when compared to the previous study. Finally, it was seen that 28% of the problematic films occurred in the morning shift, 24% occurred in the evening shift, and 48% occurred in the night shift. This data is summarized in Table 2. 

**Table 2:** Baseline quality of PCXRs within the pre-intervention cohort. PCXRs: portable chest x-rays

	Pre-Intervention
Number of total PCXRs (number of problematic PCXRs assessed)	500 (231)
Mean patient rotation score (range of 0 to 3); No Rotation (0/3), Mild Rotation (1/3), Subjective Rotation (2/3), Severe Rotation (3/3)	1.3 ± 1.0
Number of PCXRs with No Rotation (0/3)	60 (25.9%)
Number of PCXRs with Mild Rotation (1/3)	69 (29.9%)
Number of PCXRs with Subjective Rotation (2/3)	71 (30.7%)
Number of PCXRs with Severe Rotation (3/3)	31 (13.4%)
Number of PCXRs with Up/Down Rotation	100 (43.2%)
Number of PCXRs with External or Foreign Objects Obscuring Anatomy	116 (50.2%)
Number of PCXRs with Partial or Complete Anatomy Absent	115 (49.8%)
Number of studies available for comparison (within one week of PCXR)	168
Technical problem impacted ability to detect pathology when compared with previous study	73 (43.5%)
Percentage of problematic films in morning shift (8:00 AM to 3:59 PM)	28%
Percentage of problematic films in evening shift (4:00 PM to 11:59 PM)	24%
Percentage of problematic films in night shift (12:00 AM – 7:59 AM)	48%

**Figure 1:**
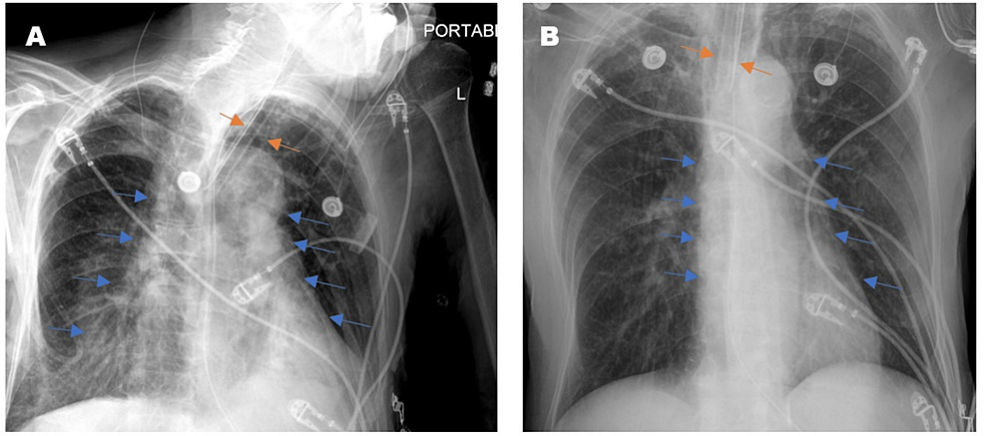
Two PCXRs from a single floor patient taken at different times of the same day. The rotated film (A) grossly exaggerates the cardiomediastinal silhouette (blue arrows) and alters the appearance of endotracheal tube positioning (orange arrows). PCXRs: portable chest x-rays

**Figure 2:**
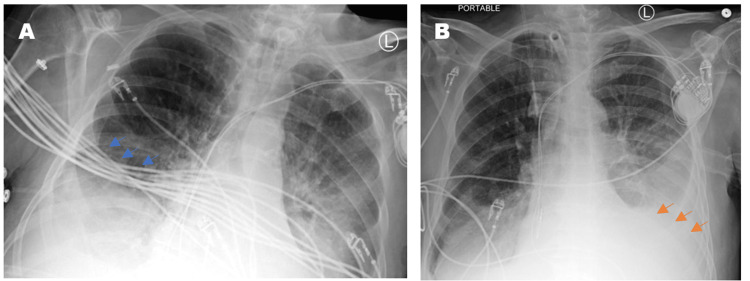
Two PCXRs from a single floor patient taken at different times of the same day. The rotated and mispositioned film (A) cuts off the left cardiophrenic angle, which ended up concealing a large left-sided pleural effusion (orange arrows). The cardiac silhouette appears exaggerated and the EKG wires (blue arrows) severely obscure evaluation of the right lower chest and the right lower lobe airspace opacities. PCXRs: portable chest x-rays

The focus group conducted involved six different technologist managers at our university hospital who presented key issues that most technologists came across when capturing PCXRs. A key issue was that some technologists did not have the physical strength to move sick and overweight patients in ICU beds, resulting in poor image quality. Improperly positioned patients can lead to severe rotations that can alter the appearance of anatomy. An example of this is shown in Figure 1, where a rotated film grossly exaggerates the cardiomediastinal silhouette and alters the appearance of the endotracheal tube positioning. Additionally, when certain machines, medical devices, and wires interfered with their ability to capture images, technologists were often unsure about how each of the medical devices could be moved appropriately. Although EKG leads could be moved easily, endotracheal tubes and chest tubes were often difficult to navigate. Accurate patient positioning was especially difficult in the ICU setting in which tubes, wires, and other ICU machinery must be repositioned to capture a clean image. This is because per policy at many institutions, technologists are not certified in manipulating biomedical devices in ICU patient beds the way nurses and physicians are. As a result, the machinery from the devices is captured in the film and appears as a foreign object covering the patient’s anatomy, which in turn, can obscure important pathological findings. An example of this can be seen in Figure 2, in which EKG wires obscured the evaluation of the right lower chest. Additionally, each patient has a unique body habitus, varying states of health, and a different past medical and surgical history. It was confirmed that each technologist takes a slightly different approach to perform the PCXR depending on the patient they are capturing images of. These slight variations in approach to position and technique can ultimately compromise image quality, impacting radiologists’ interpretation [10]. After the focus group, it was clear that more resources and support need to be allocated to the technologists so that they are better trained and supported in an increasingly demanding environment.

Three programs aiming to lower quality failure rates of PCXRs by optimizing technologist performance at our institution were proposed. First, an educational curriculum involving didactic sessions followed by practical worksheets for radiology technologists could prove beneficial. This curriculum would focus on teaching standardized strategies to improve patient positioning when obtaining PCXRs. The other two interventions were pilot programs, one where the nursing staff would assist radiology technologists with obtaining images during bedside radiography and another where internal medicine residents would verify that each PCXR film was interpretable for pathology as soon as the image had been collected before sending the films to the radiologist. The goal of these programs is to reduce the number of problematic images and to ensure that radiologists can allocate more resources towards interpreting images and diagnosing pathologies and spend less time elucidating whether an image is of high enough quality to demonstrate pathology [17]. After this meeting, it was evident that allocating more resources to help improve patient positioning was of the utmost importance to improve image quality at our institution. 

## Discussion

Historically, fixed x-rays have always been a cumbersome process, which required patients to be transferred to the radiology department by multiple staff members, even possibly increasing morbidity for seriously ill patients [18]. The advent of bedside PCXR offered optimal convenience and cost-savings for hospital systems, which explains its increase in popularity over the last two decades [18,19]. However, as workload increases for technologists taking the images, image quality decreases, which can result in poor clinical outcomes. For example, a recent study showed that poor image quality of PCXRs has raised concerns about their utility in diagnosing life-threatening conditions or monitoring of interventional line/tube placements [1,18]. Because the poor quality of fixed x-ray imaging has already been strongly linked to errors made with patient positioning during image capture [20], it is highly likely that uninterpretable PCXRs may also be due to similar errors. The purpose of our study was to quantify the baseline error rate in PCXRs at our institution, determine the potential causes of those errors, and propose various hospital-wide interventions to better support radiology technologists and reduce image quality failure rates. Analysis of 500 PCXRs over a two-week period at our single institution revealed that 231 studies were too poor in quality for accurate interpretation by a radiologist. This represents a staggering 46.2% error rate. Just this number alone further emphasizes the need for intervention to improve PCXR imaging. Of these 231 films, 28% were from the day shift, 24% from the evening shift, and 48% were from the night shift. This shows that more support needs to be allocated to the night shift as it has a significantly higher error rate than the other shifts. Out of the 231 problematic images, 168 had repeat PCXR performed within a week so that it could be determined if it were truly a technical problem that impacted the ability to detect pathology when compared to the previous study. As 43.5% of the 168 films demonstrated pathology on subsequent imaging procedures not detected during the initial imaging, we continue to demonstrate a strong need to improve imaging quality. Limitations of this study include the fact that it is a retrospective study and there may have been selection bias as only 500 PCXRs were taken in a two-week period between July 12, 2021 and July 25, 2021. Additionally, since it was a chart review, there were some missing charts that had to be discarded for the purposes of the study. Finally, there is no post-intervention data to provide further justification of the interventions proposed. 

Rationale for interventions

The first intervention proposed to improve imaging quality involved creating an educational course where technologists could learn how to appropriately position patients in a standardized fashion within our institution. This would be a longitudinal course taught by attending radiologists and senior technologists spread out over the course of three months. This intervention would compose of didactic sessions as well as worksheets where the technologists can apply the lessons learned. This intervention was selected as the most appropriate for several reasons. Firstly, patient positioning seemed to be the major source of error with PCXR film capture since nearly half of the total PCXRs assessed either had missing anatomy, foreign objects obscuring anatomy, or severe rotation. Therefore, the most straightforward solution involved additional training on the appropriate positioning of patients. The course was selected to be delivered in a longitudinal three-month period as it has been shown that longitudinal medical education courses are significantly more effective than short-term courses [21]. Mandating this educational course within the technologist department can also provide more consistency in training amongst technologists [22]. Most of the technologists are trained at different institutions with different equipment models and therefore have varying backgrounds and experience in their ability to produce quality PCXR images [23]. Therefore, the educational course would teach all technologists in the department on how to use one standardized equipment model as well as a standardized method for how the PCXRs are captured. When there is less variability in technologist performance, attending radiologists will struggle less with interpreting images because the image quality would be more consistent [22]. Having a standardized method can also allow experienced technologists to deliver more consistent and clear feedback. This is because the didactic sessions make the supervisors aware of how the department was taught, and so they can refer to specific steps of the educational process when providing feedback to each technologist [23]. This extra degree of consistency and improved communication with feedback delivery has the potential to, in turn, reduce PCXR quality errors since technologists will better understand their mistakes and prevent them from occurring in the future [23]. 

Improving organizational behavior and structure seems to be an indirect effect of implementing the didactic session. Our hospital has a large department of over 60 radiologists and 80 radiology technologists, all with varying degrees of work experience. The current literature suggests that larger department sizes can make it difficult for workers to form strong connections with one another due to the limited number of interactions they have with one another [9]. In contrast, smaller departments allow workers to speak to each other more regularly and form stronger connections with their supervisors [6,9]. Stronger interpersonal connections allow supervisors to better communicate feedback to workers in their department [9]. Although the size of our department cannot be altered, the didactic sessions can also allow newer technologists to connect with their superiors, thus promoting social interactions that would otherwise not occur. This would allow stronger connections to form and therefore foster stronger communication between workers in the department [9]. This curriculum would allow senior technologists to gain a better understanding of each worker’s baseline knowledge and skill level, which may promote more personalized feedback in the future [24]. It would also allow senior technologists to supervise newer technologists more frequently and provide more regular feedback in comparison to the sporadic feedback that newer technologists were receiving. However, senior technologists may not always be the best individuals to call upon when inexperienced technologists make sudden errors in the wards.

Often, climbing up the hospital’s administrative ladder causes a deterioration of the senior technologist’s clinical skills because they are not consistently practicing on patients [9]. The rationale for allocating nurse support to aid radiology technologists with PCXRs was so that hospital floor staff who actively practice their clinical skills can give more support and appropriate guidance to the technologists [25]. When senior technologists are called upon to address a rare mistake made by an inexperienced technologist, they may not have the knowledge or expertise to identify the error or educate their trainee due to the time spent away from clinical practice [9]. Some nurse practitioners have been trained to capture PCXRs and for those who have not, they are still regularly assisting with other forms of imaging in the hospital on a day-to-day basis, thus making their overall clinical awareness sharper than some senior technologists in some cases [26]. Therefore, nurses are more likely to observe and, hence, prevent potential mistakes being made by technologists, making their assistance valuable when PCXRs are captured in the ICU [27]. Because attending physicians and senior technologists cannot always be there to monitor each PCXR that is captured, the next best option may simply be allocating more nurses to assist. Although this means increasing responsibilities for nurses per shift, the long-term benefit could be substantial if PCXR quality errors decline, leading to less retakes of imaging.

A similar rationale was suggested for the third intervention in which internal medicine residents would verify that each PCXR film was interpretable for pathology before sending it to the radiologist. This would involve training internal medicine residents to perform a quality check of PCXR films before being passed onto the radiologist for interpretation. Residents that take a moment to check PCXR quality can potentially save technologists, nurses, patients, and attending radiologists a lot of time and resources that are currently being wasted on repeated imaging requests [28]. If the resident were to catch a poor quality PCXR early while the patient is already being imaged, the floor staff can immediately fix their error and capture another film while they are already with the patient. This is especially helpful for those extremely sick patients who may be immobile or mechanically ventilated and may not be as cooperative with imaging through the PCXR machinery. For this reason, if internal medicine residents can perform their quality checks in the ICU, where their help is needed most amongst the floor staff, it can prove highly beneficial in the consistent delivery of high-quality images to the radiologist [28]. Of note, if other hospital systems have residents from other specialties that handle their in-patient units, they can draw from these residents as well. 

## Conclusions

There is an abundance of literature that supports the philosophy that increasing educational resources and support to mammographers, ultrasound technicians, and other imaging specialists improve image quality and, therefore, the interpretive accuracy of radiologists. Although no studies currently discuss this subject in the context of PCXRs, it is quite evident that providing educational resources and support to technologists could significantly improve image quality and radiologist accuracy as well. Several interventions were proposed to provide technologists with those resources and support to ultimately capture higher-quality PCXR images. Although there is currently no available post-intervention data to assess the efficacy of these interventions, our study emphasizes the importance of creating optimal work environments for technologists through different strategies that can be implemented at the system level at other hospitals.

Future directions to our study include implementing the proposed interventions in the hospital system and evaluating a “post-intervention cohort” to see if those changes improve quality errors. Additionally, educating technologists about commonly used medical devices in the ICU is important so they feel more comfortable moving them when capturing PCXRs. Teaching technologists positioning strategies during special use cases such as specific immobilizations or heavier patients may also be of high value. Eliminating such knowledge gaps for technologists will only further boost quality and precision when they capture films for patients with complex medical conditions.
